# Molecular Characteristics of *Rickettsia* in Ticks Collected along the Southern Border of Mongolia

**DOI:** 10.3390/pathogens9110943

**Published:** 2020-11-13

**Authors:** Michael E. von Fricken, Matthew A. Voorhees, Jeffrey W. Koehler, Carmen Asbun, Brandon Lam, Barbara Qurollo, Kathryn M. Hogan, Uyanga Baasandagva, Battsetseg Jigjav, Randal J. Schoepp

**Affiliations:** 1 Department of Global and Community Health, George Mason University, Fairfax, VA 22030, USA; casbun@gmu.edu (C.A.); khogan20@gmu.edu (K.M.H.); 2 United States Army Medical Research Institute of Infectious Diseases (USAMRIID), Frederick, MD 21702, USA; matthew.a.voorhees2.civ@mail.mil (M.A.V.); jeffrey.w.koehler4.civ@mail.mil (J.W.K.); randal.j.schoepp.civ@mail.mil (R.J.S.); 3 Johns Hopkins School of Medicine, Baltimore, MD 21218, USA; blam4@jhmi.edu; 4 Department of Clinical Sciences, College of Veterinary Medicine, North Carolina State University, Raleigh, NC 27606, USA; baquroll@ncsu.edu; 5 National Center for Zoonotic Diseases, Ulaanbaatar 16000, Mongolia; uyanga@nczd.gov.mn (U.B.); nczd@nczd.gov.mn (B.J.)

**Keywords:** Mongolia, *Rickettsia*, tick-borne diseases, *Hyalomma*, *Dermacentor*, surveillance

## Abstract

Tick-borne infections are a significant threat to public health, particularly in regions where individuals frequently enter tick habitats. Roughly 26% of the population in Mongolia practice nomadic pastoralism and are considered at high risk of exposure to ticks and the diseases they carry. This study tested ticks from Mongolia’s southern border for *Rickettsia* spp. to better understand the epidemiology of tick-borne diseases in the region. *Dermacentor nuttalli* and *Hyalomma asiaticum* ticks (*n* = 4022) were pooled and tested for *Rickettsia* spp. by real-time PCR. Melt-curve analyses and Sanger sequencing were used to identify *Rickettsia* species. Approximately 64% of the 786 tick pools tested positive for *Rickettsia* bacteria. Melt curve analyses identified four different *Rickettsia* species circulating in these tick pools. Amplicon sequencing of the *ompA* gene identified *Rickettsia* spp. that closely resembled *R. raoultii* and *R. sibirica*. *Dermacentor nuttalli* ticks from Govi-Altai had the highest maximum likelihood estimation infection rate 48.4% (95% CI: 41.7–56.5%), while *Hyalomma* *asiaticum* collected in Omnogovi had a rate of 7.6% (95% CI: 6.2–9.2%). The high detection of *Rickettsia* suggests a substantial risk of infection in southern Mongolia. Further studies are necessary to investigate the clinical burden of tick-borne diseases in Mongolia.

## 1. Introduction

Rickettsial infections are on the rise globally and pose an emerging threat to human health [1,2,3]. Transmission and infection occur after arthropods such as ticks and fleas suck blood from a host, causing mild to fatal illnesses (ex. spotted fevers and typhus) characterized by non-specific fever, myalgia nausea, and rash [4]. Spotted fever group (SFG) rickettsial organisms are gram-negative obligate intracellular coccoid-shaped bacteria that can infect a variety of mammalian species, including livestock and humans. Tick-borne SFG *Rickettsia* are distinguished from the *Rickettsia* typhus group (TG) by vector, clinical presentation, and the presence of the outer membrane protein *ompA*, which is absent in the TG *Rickettsia* [5]. The epizootiology of *Rickettsia* spp. is further complicated by transovarial and transstadial transmission within the vector tick species [6].

Roughly 26% of Mongolia’s population of three million residents engage in traditional pastoral herding. This subset of the population is at an increased risk of exposure to both zoonotic and vector-borne infectious diseases [7,8,9]. Tick-borne rickettsioses in particular have a significant impact on the health of this at-risk population: peak tick bite rates occur during economically productive months (increased risk of disease), and low healthcare-seeking rates despite the presence of symptoms delay treatment [10]. Tick-borne diseases result in additional economic losses incurred from illness in livestock [8].

Multiple Rickettsia species, including R. raoultii, R. sibirica sibirica, R. sibirica mongolitimonae, R. heilongjiangensis, and “Candidatus R. tarasevichiae”, have been identified in Dermacentor spp., Ixodes persulcatus, and Haemaphysalis concinna ticks collected in and around Mongolia [3,8,11,12,13,14]. Of these, only R. raoultii (scalp eschar and neck lymphadenopathy after tick bite, or SENLAT) and R. sibirica sibirica (Siberian tick typhus) are known to cause human disease [15]. Characterization of Rickettsia species is also important due to serious clinical diseases in neighboring countries [16,17,18,19,20,21]. A previous study by our group collected and tested Dermacentor nuttalli and Hyalomma asiaticum tick pools for the presence of Crimean-Congo hemorrhagic fever virus by real-time RT-PCR [9]. Here, we utilized this same sample set to assess the presence and distribution of different Rickettsia spp. in Mongolia.

## 2. Results

A total of 4022 ticks [*Dermacentor nuttalli* (*n* = 2396) and *Hyalomma asiaticum* (*n* = 1626)] were collected across southern Mongolia. Livestock sampling resulted in the collection of 592 *D. nuttalli* (25.7%) and 1028 *H. asiaticum* (63.2%) partially fed ticks, with the remaining 2402 ticks collected directly from the environment. A total of 467 *D. nuttalli* and 319 *H. asiaticum* tick pools, sorted by collection method and location, were included for testing. Initial testing by real-time PCR found 64% of tick pools tested positive (505/786) for *Rickettsia* spp. with *D. nuttalli* and *H. asiaticum* detection rates of 86% and 33%, respectively. The highest *Rickettsia* spp. pool detection rate was observed in Govi-Altai at 95% (195/204). Table 1 depicts a summary of the tick collection locations, species identification, and testing results.

**Figure 1:**
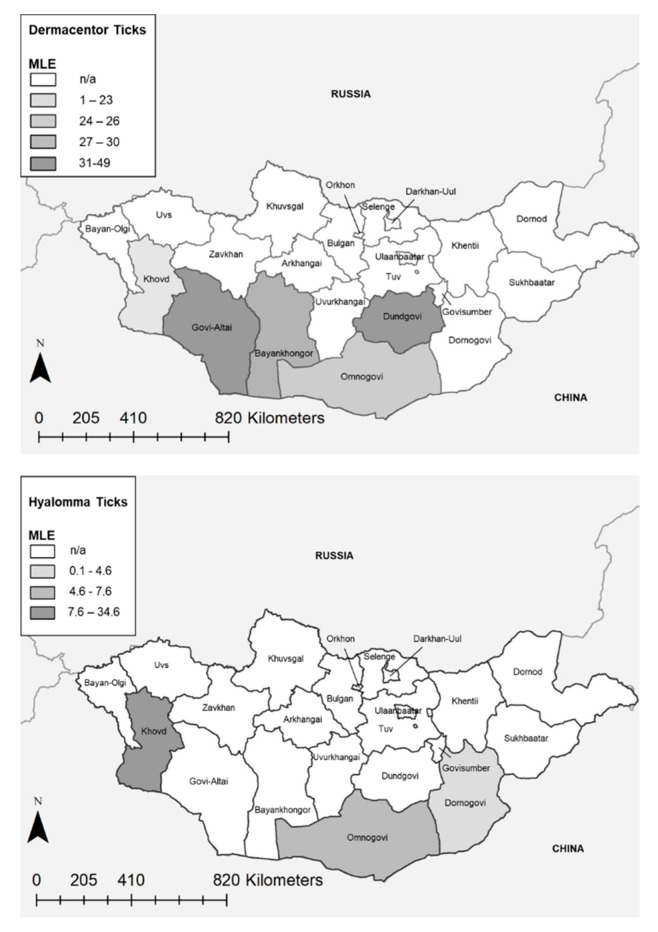
Maximum likelihood estimates (MLE) by Aimag for *Dermacentor* (**above**) and *Hyalomma* (**below**) ticks.

Overall, maximum likelihood estimates (MLE) found *Rickettsia* spp. present at an average prevalence of 33.2% (95% CI: 30.1–36.2%) in *Dermacentor* ticks, and 7.4% (95% CI: 6.1–8.9%) *Hyalomma* ticks collected along the southern border (Figure 1). Bayankhongor had an 82% prevalence (55/67 positive tick pools) with an MLE of 30.1% (95% CI: 22.9–37.3%) and a minimum infection rate (MIR) of 16.5% (95% CI: 12.5–20.4%). In contrast, Omnogovi aimag had the highest percentage of *Rickettsia*-positive *Hyalomma* ticks, with a pool-positive rate of 33% (96/289), an MLE of 7.59% (95% CI: 6.24–9.16%), and MIR of 6.5% (95% CI: 5.2–7.6%). No association was observed in detection rates when examining *H. asiaticum* (*p =* 0.48) and *D. nuttalli* (*p =* 0.27) removed from livestock, compared to those collected from the environment.

**Figure 2:**
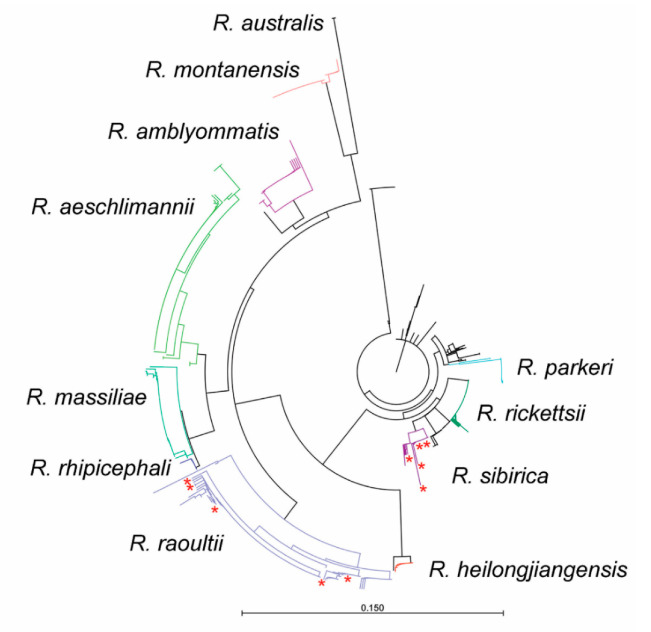
Sequence analysis of *ompA* gene fragment. *Rickettsia ompA* sequences from the Mongolian tick samples (stared, in red) were aligned with *ompA* sequences from multiple *Rickettsia* species found in GenBank. A phylogenetic tree (Neighbor Joining, Jukes-Cantor) highlights the genetic diversity of the detected *Rickettsia* species.

Melt curve analysis of the amplicon generated at the end of the real-time PCR reaction can differentiate some species of *Rickettsia* based on the impact nucleic acid compositional differences have on strand binding kinetics. Analysis of these melt curves identified at least eight distinct curves, suggesting a wide diversity of *Rickettsia* spp. in circulation in the Mongolian tick population. Thirty samples were selected for Sanger sequencing based on the melt curve analysis, tick species, and geographic distribution (Supplementary Table S1). An approximate 212 base pair segment of *ompA* was amplified, sequenced, and BLAST-identified. *Rickettsia raoultii* (*n* = 13)*, R. sibirica mongolitimonae* (*n* = 6), and *R. sibirica* (*n* = 2) were detected. One isolate from Dornogovi, located in southeastern Mongolia, shared 97.66% identity with *R. sibirica mongolitimonae* (MK922654) and was detected in a pool of *H. asiaticum* ticks removed from livestock (Figure 2).

## 3. Discussion

*Rickettsia* spp. circulate at a high rate within native tick species in Mongolia, presenting a significant health risk to pastoralist populations in close contact with their environment. The highest MLE rate of 48.4% (95% CI: 41.7–56.5%) was observed in *Dermacentor* ticks from the Govi-Altai region. Additionally, an MLE rate of 7.6% (95% CI: 6.2–9.2%) was observed in *Hyalomma* ticks collected in Omnogovi, warranting further testing. Overall, a large percentage of *D. nuttalli* pools (86%) tested positive for *Rickettsia* spp. by real-time PCR, and nearly all the ticks tested from the Govi-Altai region tested positive. Melt curve analysis found a high amount of *Rickettsia* spp. diversity; *ompA* sequencing identified four species of *Rickettsia* (*R. raoultii*, *R. sibirica mongolitimonae*, *R. sibirica,* and one species closely related to *R. sibirica mongolitimonae*) known to cause human disease. Utilizing melt curve analysis in tandem with Sangar sequencing allowed our team to detect multiple circulating *Rickettsia* species without requiring extensive sequencing of positive samples.

Speck and colleagues (2012) found prevalence rates of *R. raoultii* (82%) and *R. sibirica* (4%) in *Dermacentor nuttalli* ticks in northern Mongolia; 5% of the identified *Rickettsia* spp. were not able to be assigned to a specific tick species [14]. Most infected ticks were found in the Selenge and Khentii aimags, with *R. sibirica* being found exclusively in ticks from Arkhangai aimag [14]. PCR analysis of ticks collected at Sino-Russian and Sino-Mongolian borders found a 53.4% prevalence of *Rickettsia* phylogenetically belonging to *R. raoultii* [20]. Boldbaatar and colleagues (2017) detected a 15.1% prevalence of *R. raoultii* in *D. nuttalli* in Dornogovi (a southern aimag), while higher maximum likelihood estimates (MLEs) were found in the northern aimags of Selenge and Tov [7]. This study found an MLE of 39.6 (95% CI: 17.4–61.3%) in Dornogovi among *Dermacentor* ticks; however, this finding is limited given the sample size (12 pools). *Hyalomma* ticks collected from the same area had a much lower MLE, 4.6 (95% CI: 2.1–9.2%), with only 6/27 pools testing positive. Furthermore, previous work in Dornogovi has indicated serological evidence of *Rickettsia* exposure in both herders and livestock [8]. The high detection rates observed in this study, paired with previously published findings from elsewhere in Mongolia, indicate that the distribution of ticks harboring pathogenic *rickettsia* is ubiquitous throughout the country, representing a major public health concern.

Continued vector surveillance is necessary, especially in the Govi-Altai region, which had the highest detection rate in sampled ticks for this study. Enhanced serological and syndromic surveillance are also needed to determine the clinical burden of SFG *Rickettsia* in Mongolia, paying particular attention to pastoral herding populations. Such studies will help characterize the relationship between the high detection rates of *Rickettsia* found in Mongolian ticks and their impact on both human and animal health. Considering the severity of the clinical symptoms of *Rickettsia* isolates reported in neighboring China and Russia, it is possible that these same pathogens may also be circulating in Mongolia.

## 4. Materials and Methods

### 4.1. Sample Collection, Study Location, and Processing

Questing environmental ticks and ticks removed from livestock were collected in 2013 and 2014 by the National Center for Zoonotic Diseases (Ulaanbaatar, Mongolia) from five aimags in southern Mongolia (Khovd, Govi-Altai, Bayankhongor, Omnogovi, and Dornogovi;). Adult *Dermacentor nuttalli* (*n* = 2396) and *Hyalomma asiaticum* (*n* = 1626) ticks were pooled into 2011 pools based on tick identity, sex, geographic location, and collection method (livestock vs. environment). Tick species were determined based on morphological classification, using local keys. Tick pools were homogenized (SPEX SamplePrep MiniG^®^ 1600 tissue homogenizer (Metuchen, NJ, USA)) and total nucleic acid extracted (TRIzol LS^®^ reagent, KingFisher Flex Purification System, MagMax 96 for MicroArrays Total RNA Isolation Kit (ThermoFisher Scientific, Waltham, MA, USA)). These homogenates were further pooled for testing (786 pools of 2–6 ticks each). All extracted nucleic acid and homogenized tick pools were stored at −70 °C until testing.

### 4.2. Rickettsia spp. Testing

Five microliter nucleic acid pools were tested in duplicate for *Rickettsia* spp. utilizing a real-time PCR assay with melt curve analysis targeting the 23s–5s ITS region with 0.4 μM (final concentration) primers Rick23-5 F (5′- AGCTCGATTGATTTACTTTGCTG -3′) and Rick23-5 R (5′- CCACCAAGCTAGCAATACAAA-3′) and SsoAdvanced SYBR Green Supermix (Bio-Rad, Hercules, CA, USA) in a 25 μL reaction [22]. Cycling conditions were 98 °C for 3 min; 40 cycles of (98 °C for 15 s, 62 °C 15 s, and 72 °C for 15 s) were followed by a melt curve analysis of the 75–90 °C range with measurements in 0.5 °C increments. Samples were run on the LightCycler 480 (Roche, Indianapolis, IN, USA). Melt curve analysis was used as a rationale to identify candidates for sequencing, based on amplicon melt temperatures potentially indicating different *Rickettsia* spp.: *R. amblyomma* (78 °C), *R. bellii* (76.5 °C), *R. canadensis* (77.5 °C), *R. conorii* (77.5 °C), *R. montanesis* (77 °C), *R. parkeri* (78 °C), *R. typhi* (75.5 °C), *R. rickettsii* (77 or 78 °C), *R. rhipicephali* (78 °C), *R. felis* (78 °C), “*Candidatus* R. amblyommii” (78.5 °C), *R. honei* (78 °C), and *R. raoultii* (78 °C) [22].

A 212 base pair amplicon of *ompA* was amplified and sequenced using the Big Dye Direct Sanger Sequencing Kit (ThermoFisher Scientific) for samples selected based on the melt curve analysis. Amplification used the primers Rick-ompA-F (5′-TGTAAAACGACGGCCAGTGCTTTATTCACCACCTCAAC) and Rick-ompA-R (5′- CAGGAAACAGCTATGACCTRATCACCACCGTAAGTAAAT) modified for the Big Dye Direct Sanger Sequencing kit (underlined sequence). Sequences from the forward and reverse primers were assembled and analyzed using CLC Genomics Workbench v10.1.2. (Qiagen, Hilden, Germany) Amplicon sequences, not including the primer sequences, were deposited into GenBank (#MW013059-MW013079).

### 4.3. Statistical Analysis

Maximum likelihood estimates (MLE) and minimum infection rates (MIR) were calculated to estimate the likelihood of pathogen detection from pooled samples based on laboratory findings, both of which are common measurements used when examining pooled samples. Chi-square statistic was used to determine significance (*p* < 0.05) in *Rickettsia* detection rates by species between ticks removed from livestock and those collected from the environment.

## 5. Conclusions

Rickettsial pathogens have a complex disease ecology, with a wide distribution of hosts that includes mammals, humans, and ectoparasites. Public health campaigns can be used to increase awareness and inform populations within these regions of the potential risk ticks play, especially among pastoralist who have regular contact with livestock. This study found an alarming proportion of adult ticks carrying bacterial species belonging to the SFG *Rickettsia*. Increased syndromic surveillance, particularly in southern Mongolia and among high-risk populations, is needed to further characterize the epidemiology of tick-borne diseases in Mongolia.

## Figures and Tables

**Table 1:** Maximum likelihood estimates and minimum infection rate by region based on qPCR results, including 95% confidence intervals.

Province	Genus	Positive Pools (%)	Total Ticks	MLE			MIR		
				Point	Low	High	Point	Low	High
Bayankhongor	*Dermacentor*	55/67 (82%)	334	30.1	22.9	37.3	16.5	12.5	20.4
Dornogovi	*Dermacentor*	10/12 (83%)	58	39.6	17.4	61.3	17.2	7.5	27.0
Govi-Altai	*Dermacentor*	195/204 (96%)	1058	48.9	41.2	55.8	18.4	16.1	20.8
Khovd	*Dermacentor*	34/46 (74%)	238	23.2	16.4	30.4	14.3	9.8	18.7
Omnogovi	*Dermacentor*	107/138 (78%)	708	26.9	21.6	30.8	15.1	12.5	17.8
Dornogovi	*Hyalomma*	6/27 (22%)	144	4.6	2.1	9.2	4.2	1.0	7.4
Khovd	*Hyalomma*	2/3 (66%)	10	34.6	7.3	69.7	20.0	0.0	44.8
Omnogovi	*Hyalomma*	96/289 (33%)	1472	7.6	6.2	9.2	6.5	5.2	7.8
Total	*Dermacentor*	401/467 (86%)	2396	33.2	30.1	36.2	16.7	15.2	18.2
Total	*Hyalomma*	104/319 (33%)	1626	7.4	6.1	8.9	6.4	5.2	7.6

## Supplementary Materials

The following are available online at https://www.mdpi.com/2076-0817/9/11/943/s1, Table S1: *ompA* sequences and accession numbers.
